# MiR-216a-5p targets TCTN1 to inhibit cell proliferation and induce apoptosis in esophageal squamous cell carcinoma

**DOI:** 10.1186/s11658-019-0166-9

**Published:** 2019-06-28

**Authors:** Lixun Chai, Gengpu Yang

**Affiliations:** Department of Thoracic Surgery, Shanxi Dayi Hospital, No. 99 Dragon City Street, Taiyuan, 030012 Shanxi Province China

**Keywords:** ESCC, miR-216a-5p, TCTN1, Proliferation, Apoptosis

## Abstract

**Background:**

MiR-216a-5p has been reported to be associated with several tumors, including prostate cancer and melanoma. However, its expression level and potential role in esophageal squamous cell carcinoma (ESCC) remain uncertain.

**Results:**

Here, we found that miR-216a-5p expression was significantly down-regulated in clinical ESCC tissues and cells. Functional assays were performed to evaluate the biological effects of miR-216a-5p on cell proliferation and cell apoptosis by CCK-8 assay and flow cytometry in ESCC cell lines, EC9706 and TE-9. The results showed that miR-216a-5p overexpression repressed cell proliferation and induced cell apoptosis. Through bioinformatics prediction and luciferase reporter assay, we revealed that miR-216a-5p could directly target tectonic family member 1 (TCTN1). Moreover, TCTN1 was obviously suppressed by miR-216a-5p overexpression. In addition, TCTN1 expression was significantly increased and inversely correlated with the levels of miR-216a-5p in ESCC tissues. More importantly, down-regulation of TCTN1 imitated, while restoration of TCTN reversed the effects of miR-216a-5p on cell proliferation and apoptosis. At the molecular level, we further found that TCTN1 overexpression reversed the effects of miR-216a-5p transfection on the expression of PCNA, Bcl-2 and Bad.

**Conclusions:**

Our results demonstrate that miR-216a-5p might serve as a tumor suppressor in ESCC cells through negatively regulating TCTN1 expression, indicating the possibility that miR-216a-5p and TCTN1 might be attractive targets for ESCC therapeutic intervention.

## Background

Esophageal squamous cell carcinoma (ESCC) ranked as the sixth most commonly diagnosed digestive system cancer in the United States in 2017 [1]. It is estimated that there are 16,940 new cases (13,360 in men and 3580 in women) and 15,690 deaths (12,720 in men and 2970 in women) each year in the United States [1]. Several environmental and/or genetic factors, including obesity, alcohol, and tobacco, were considered to be associated with the etiology of ESCC [2, 3]. Although some progress has been made in the studies, including changes in diet and physical activity, the survival prognosis still remains poor [2, 4, 5]. It is notable that a deep understanding of the molecular mechanisms underlying the biological behavior of ESCC will provide important clues for improvement of survival and life quality in patients.

MicroRNAs (miRs) are endogenous small non-coding RNAs that play a variety of diverse roles in biology, including cell cycle progression, proliferation, apoptosis, development and differentiation [6, 7]. By binding to the 3′-untranslated region (UTR) of their target mRNAs, miRNA can specifically cause protein expression reduction predominantly by destabilizing the target mRNAs and/or by repressing translation [8] [8, 9]. It now appears that a number of miRs are dysregulated in multiple cancers and involved in various pathological and physiological conditions in cancer cells [9, 10]. Some miRs were found to be upregulated or down-regulated in ESCC, which are thought to play key roles in tumorigenesis and development, such as miR-644a [11], miR-130b [12], and miR-375 [13]. Although the biological role of only a limited number of miR transcripts has been identified in ESCC, many miRs are still unknown.

As a newly identified miRNA encoded by the chromosomal region 2q16.1, miR-216a-5p has received growing attention in recent years [14]. It is reported that miR-216a-5p is down-regulated in pancreatic tissues, and exerts inhibitory effects on proliferation, migration, and invasion in pancreatic ductal adenocarcinoma cells [14]. With regards to lung cancer, the levels of miR-216a-5p are lower in tumor tissues compared with normal lung tissues, and act as an anti-oncogene in lung cancer [15]. Using a synthesized miR-216a-5p inhibitor, decrease of miR-216a-5p inhibited cell viability and motility, thereby depressing tumor growth in renal cell carcinoma cells [16]. However, the role of miR-216a-5p in ESCC remains undefined.

Tectonic family member 1 (TCTN1) involved in the human Hedgehog signaling pathway is a member of tectonic transmembrane proteins [17]. In human endothelial cells, TCTN1 was found to promote endothelial nitric oxide synthase [18]. TCTN1 is also a known protein component of a ciliopathy-associated protein complex and could interact with Mks1, Tmem216, Tmem217, and several other proteins that are associated with ciliopathies to modulate ciliary assembly and trafficking [19]. Interestingly, the primary cilium has an important role in cell-to-cell communication by sensing Hedgehog and Wingless signaling pathways, whose dysregulation affects cancer development, tumorigenesis and prognosis [20–22]. Recent studies revealed that TCTN1 is widely up-regulated in various types of human cancer, including gastric cancer [23], colorectal cancer [24], prostate cancer [25], and glioblastoma [26] and acts as an oncogene via promoting proliferation, migration, or inhibiting apoptosis.

In the present study, we aimed to determine levels of miR-216a-5p expression in human ESCC tissues and cell lines. Then, the role of miR-216a-5p in ESCC was investigated by gain-of-function experiments. We focused on TCTN1 as a candidate target of miR-216a-5p, as it was predicted by TargetScan. It is certain that understanding the association between miR-216a-5p and TCTN1 could help us develop a useful therapeutic target for ESCC treatments.

## Materials and methods

### Clinical specimens

In total, 25 pairs of human ESCC tissues and matched adjacent tissues (> 5 cm away from the tumor margin) were collected from 25 patients who underwent esophagus resection without chemotherapy or radiotherapy at the Department of Thoracic Surgery, Shanxi Dayi Hospital (Shanxi, China) between January 2017 and March 2018. All the collected samples were immediately snap-frozen in liquid nitrogen and kept at − 80 °C for RNA extraction. The basic clinicopathologic characteristics of all patients are listed in Table 1. Written informed consent was obtained from all participants.

**Table 1:** Clinicopathological characteristics of the esophageal squamous cell carcinoma patients (*n* = 25)

Patient characteristics	Cases (*n* = 25)
Gender	
Male	16
Female	9
Age	
< 60	19
≥ 60	6
Tumor size (cm)	
< 3	18
≥ 3	7
TNM stage	
I/II	21
III/IV	4
Differentiation	
Well	8
Moderate	12
Poor	5

*TNM* Tumor node metastasis

### Cell culture and transfection

Human ESCC cell lines (KYSE150, EC9706, KYSE30 and TE-9) and esophageal epithelial cells (HET-1A) were obtained from the Chinese Academy of Science cell bank (Shanghai, China) and cultured in Roswell Park Memorial Institute-1640 medium (RPMI-1640; Invitrogen; Thermo Fisher Scientific, Inc., USA) containing 10% fetal bovine serum (FBS, Gibco; Thermo Fisher Scientific, Inc.). All cell lines were maintained in a humidified atmosphere with 5% CO_2_ at 37 °C.

The synthesized miR-216a-5p mimics (miR-216a-5p), miR-216a-5p inhibitor (inhibitor), negative control (miR-NC), small interfering RNA for TCTN1 (siTCTN1) and its NC (siNC) were purchased from Shanghai GenePharma Co., Ltd. (Shanghai, China). MiR-216a-5p overexpression was accomplished by transfecting EC9706 and TE-9 cells with 0.1 μM miR-216a-5p mimics or miR-NC for 48 h. MiR-216a-5p silencing was achieved by transfecting HET-1A cells with 0.1 μM inhibitor or miR-NC for 48 h. For TCTN1 silencing, EC9706 and TE-9 cells were transfected with siTCTN1 or siNC at a final concentration of 50 nM for 48 h. TCTN1 coding sequences were sub-cloned into pcDNA3.1 (Sangon Biotech, China) to construct the TCTN1 overexpression vector (TCTN1). The empty vector was used as a negative control. In the rescue experiments, EC9706 cells were co-transfected with miR-216a-5p or miR-NC together with TCTN1 or the empty vector. All cell transfections were carried out using Lipofectamine 2000 (Invitrogen, Waltham, MA, USA) in accordance with the manufacturer’s instructions.

### RNA extraction and quantitative real-time PCR (qRT-PCR)

Total RNA was extracted from tissues or cells using Trizol reagent (Invitrogen, MA, USA), 1 μg RNA of which was used for reverse transcription using PrimeScript RT Reagent (Takara Bio, Inc.). The expression of miR-216a-5p and TCTN1 was measured using a miScript SYBR-Green PCR kit (Takara Bio, Inc.) and SYBR Premix Ex Taq (Takara Bio, Inc.), respectively. All qRT-PCR reactions were performed on an ABI PRISM 7300 Fast Real-Time PCR System (Ambion, Foster City, CA, USA) with the following thermocycling conditions: 95 °C for 1 min, 40 cycles of 95 °C for 15 s, 55 °C for 30 s and 72 °C for 30 s. The primer sequences used were as follows: miR-216a-5p, 5′-TGTCGCAAATCTCTGCAGG-3′ (forward) and 5′-CAGAGCAGGGTCCGAGGTA-3′ (reverse); U6, 5′-CTCGCTTCGGCAGCACA-3′ (forward), and 5′-ACGCTTCACGAATTTGCGT-3′ (reverse); TCTN1, 5′-CCTTTGCGTGAATGTTGTTC-3′ (forward), and 5′-AGAGGGACTGGCTGGGTATT-3′ (reverse); GAPDH, 5′-GCACCGTCAAGGCTGAGAAC-3′ (forward), and 5′-TGGTGAAGACGCCAGTGGA-3′ (reverse). The relative expression of miR-216a-5p or TCTN1 was determined by the 2^−ΔΔCq^ method. U6 and GAPDH were used as an internal control for miR-216a-5p and TCTN1, respectively.

### Cell proliferation assay

ESCC cells transfected with miR-216a-5p or siTCTN1 were collected and seeded into 96-well plates at a density of 3 × 10^3^ cells per well. Subsequently, 10 μL of CCK-8 assay solution (Dojindo Laboratories, Kumamoto, Japan) was added to each well at the indicated time points and cells were incubated for 1 h at 37 °C. Using a microplate reader, cellular proliferation was measured by detecting the absorbance at 450 nm.

### Flow cytometry assay

The cell apoptosis were assessed by a flow cytometer (BD FACSCalibur; BD Biosciences) with double Annexin V/PI staining (Invitrogen). In brief, approximately 3 × 10^5^ transfected cells were harvested from a 6-well plate by centrifugation and mixed with 500 μl of 1X binding buffer, followed by staining with 5 μl of FITC-Annexin V and propidium iodide (PI). The early apoptotic (Annexin V+/PI-) and late apoptotic (Annexin V+/PI+) cells were analyzed by flow cytometry and the total apoptotic rate was calculated in each group.

### Dual luciferase reporter assay

TargetScan (http://www.targetscan.org/vert_71/) was applied to predict the putative targets of miR-216a-5p. To confirm whether miR-216a-5p directly targets the 3′-UTR of TCTN1, the wild-type or mutant 3′-UTR of TCTN1 was amplified and cloned into the vector psiCHECK-2 to construct luciferase reporter plasmids (WT TCTN1 or MUT TCTN1, respectively). For the luciferase reporter assay, 293 T cells (1 × 10^4^/well) were co-transfected with WT TCTN1 or MUT TCTN1 together with miR-216a-5p or miR-NC using Lipofectamine 2000 according to the manufacturer’s instructions. After 48 h of transfection, the Renilla and firefly luciferase activity was detected using the Dual-Luciferase Reporter Assay kit (Promega Corp., Madison, WI, USA). Data were expressed as the ratio of Renilla luciferase activity normalized to Renilla luciferase activity.

### Western blotting analysis

Total proteins were extracted from cells using lysis buffer (Beyotime Institute of Biotechnology, Haimen, China) and quantified according to the protocol of a BCA Protein Assay Kit (Thermo Fisher Scientific). Equal amounts of protein samples were subjected to separation on 10% SDS-PAGE gels and transferred onto polyvinylidene fluoride (PVDF) membranes (Millipore, Billerica, MA, USA). Then, the membranes were blocked with 5% skim milk for 2 h at room temperature, followed by incubation with primary antibodies against TCTN1, PCNA, Bcl-2, Bad and GAPDH overnight at 4 °C. After incubation with horseradish peroxidase-conjugated secondary antibody for 2 h, the membranes were visualized using an ECL kit (Beyotime Institute of Biotechnology, Beijing, China).

### Statistical analysis

All statistical analyses were performed using GraphPad Prism 6.0 (GraphPad Software, Inc., La Jolla, CA, USA) and data were expressed as the mean ± standard deviation (SD). The correlation between miR-216a-5p expression and TCTN1 mRNA expression levels was examined using Pearson’s correlation analysis. Comparisons between two groups were made using Student’s t-test. Differences among multiple groups were assessed using one-way analysis of variance followed by Tukey’s test. The value of *p* < 0.05 was considered to be statistically significant.

## Results

### MiR-216a-5p was significantly downregulated in ESCC tissues and cell lines

**Fig. 1:**
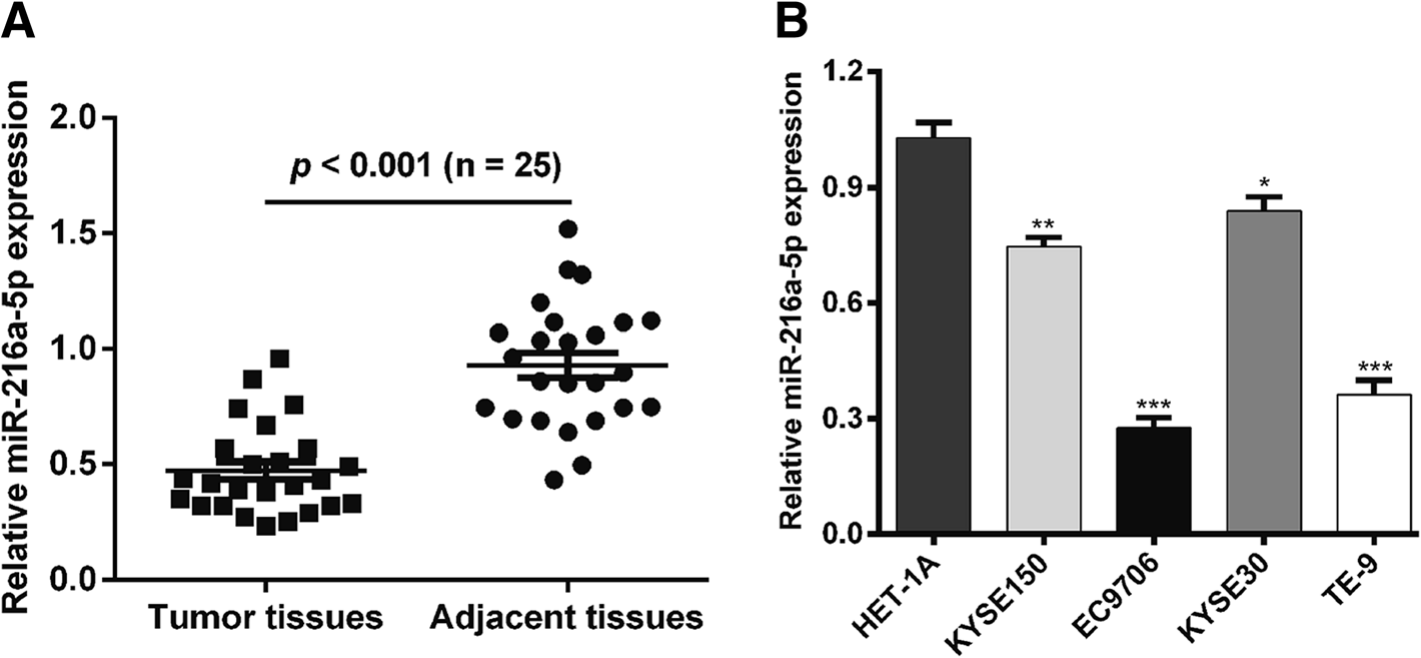
MiR-216a-5p was significantly down-regulated in ESCC tissues and cell lines. **a** MiR-216a-5p expression levels in 25 pairs of ESCC tissues compared with matched adjacent normal tissues were determined using qRT-PCR. **b** MiR-216a-5p expression in four ESCC cell lines and normal esophageal epithelial HET-1A cells was measured using qRT-PCR. *p < 0.05, **p < 0.01, ***p < 0.001 vs. normal group; ESCC, esophageal squamous cell carcinoma; qRT-PCR, quantitative real-time PCR

To explore the functional role of miR-216a-5p in ESCC, the expression levels of miR-216a-5p were determined in ESCC tissues and the adjacent non-tumor tissues by qRT-PCR. As illustrated in Fig. 1a, miR-216a-5p was significantly downregulated in 25 pairs of ESCC tissues in comparison with matched adjacent tissues. The miR-216a-5p expression levels were also measured in four human ESCC cell lines: KYSE150, EC9706, KYSE30 and TE-9. The results revealed that miR-216a-5p was also markedly decreased in all four ESCC cell liens compared with those in normal esophageal epithelial HET-1A cells (Fig. 1b). Among the four ESCC cell lines, EC9706 and TE-9 expressed the lowest miR-216a-5p levels and were thus selected for further analyses.

### MiR-216a-5p suppressed ESCC cell proliferation and promoted cell apoptosis

**Fig. 2:**
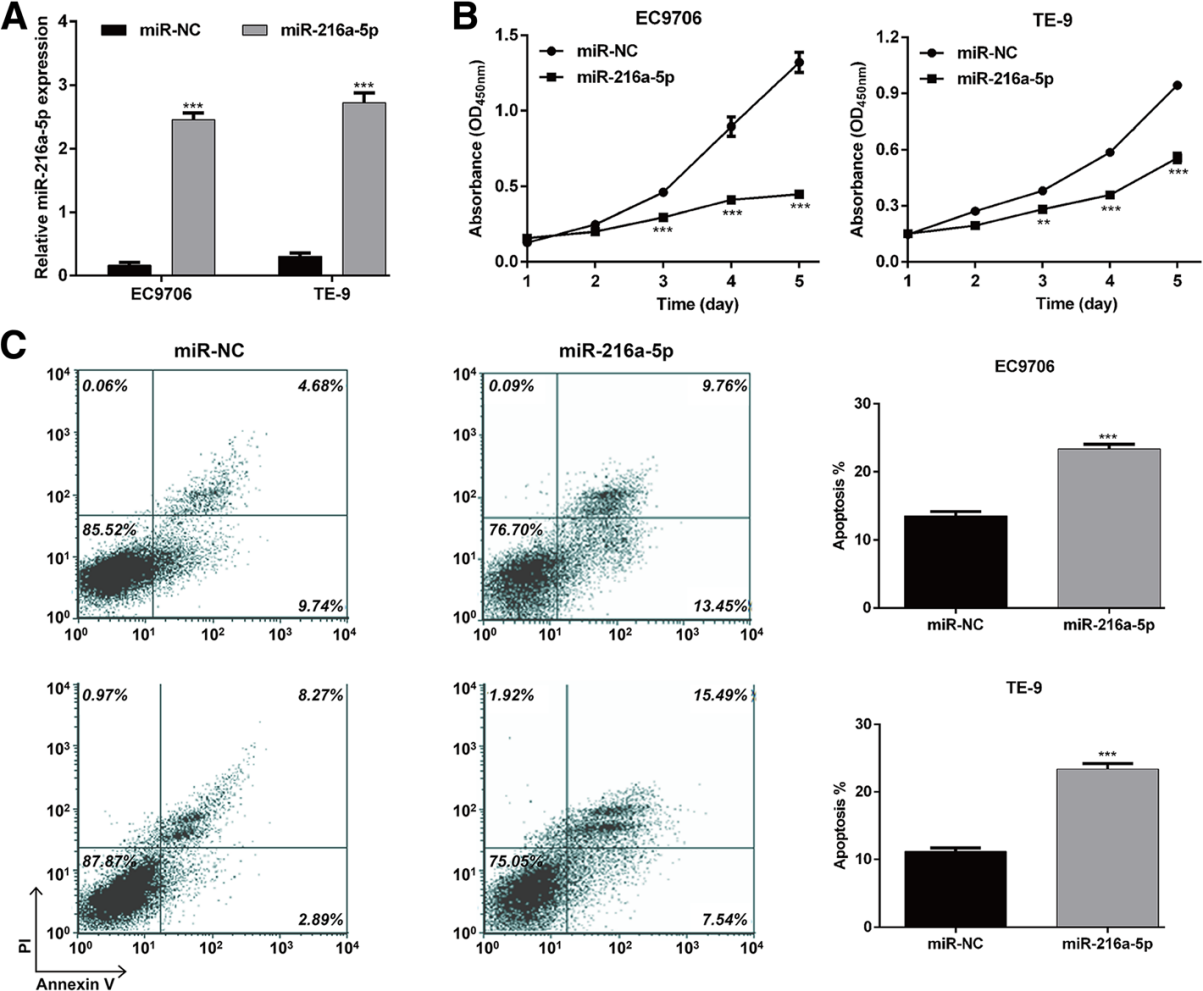
MiR-216a-5p inhibited cell proliferation and induced apoptosis in ESCC cells. **a** qRT-PCR was used to determine miR-216a-5p in EC9706 and TE-9 cells following transfection with miR-216a-5p or miR-NC. **b** CCK-8 assay was used to analyze the effect of miR-216a-5p overexpression on ESCC cell proliferation. **c** Flow cytometry with double Annexin V/PI staining was used to detect the effect of miR-216a-5p overexpression on ESCC cell apoptosis. ***p < 0.001 vs. miR-NC

We then investigated the biological function of miR-216a-5p by transfecting miR-216a-5p mimics or miR-NC in EC9706 and TE-9 cells, which exhibited low expression of miR-216a-5p. Subsequent to transfection, qRT-PCR confirmed that miR-216a-5p was significantly increased in EC9706 and TE-9 cells after miR-216a-5p mimic transfection (Fig. 2a, *p* < 0.001). The results of CCK-8 assay revealed that miR-216-5p overexpression dramatically suppressed the proliferation of EC9706 and TE-9 cells compared with the miR-NC group (Fig. 2b, *p* < 0.001). Moreover, cell apoptosis assays were performed in the miR-216a-5p transfected cells by flow cytometry. As shown in Fig. 2c, the overall apoptotic percentage (EC9706: 13.54% ± 0.63% vs. 23.38% ± 0.67%; TE-9: 11.15% ± 0.54% vs. 23.39% ± 0.84%) in the miR-216a-5p group was significantly higher than that in the miR-NC group. These results demonstrated that miR-216a-5p might act as a tumor suppressor in ESCC by inhibiting cell proliferation.

### TCTN1 is a direct target of miR-216a-5p in ESCC

**Fig. 3:**
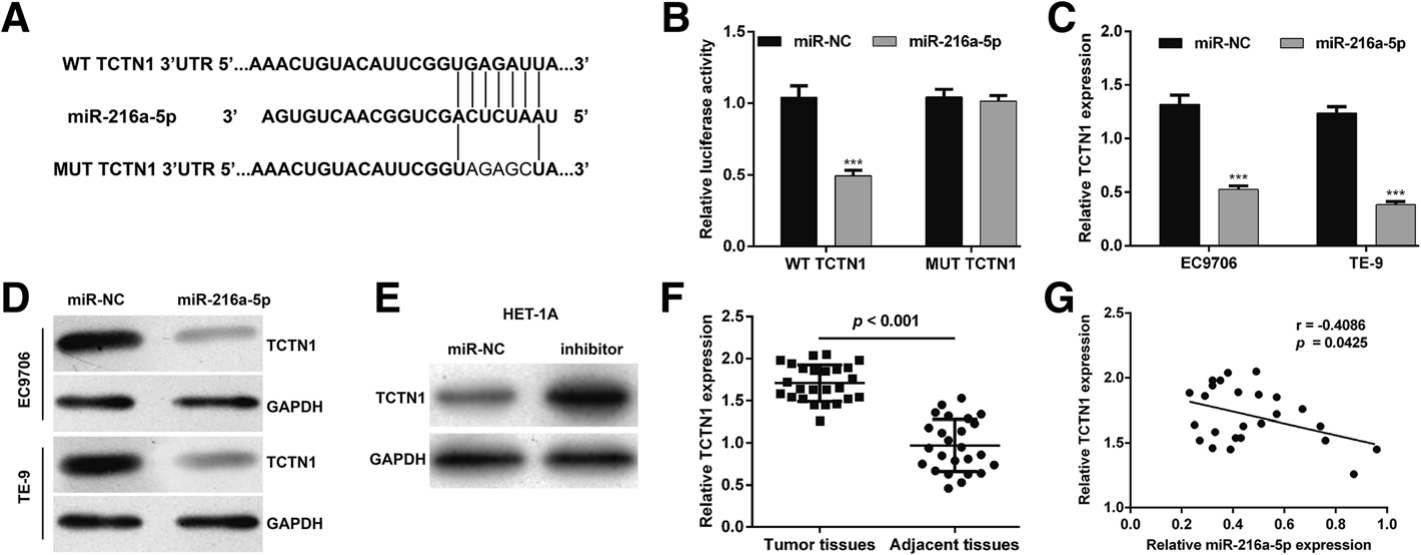
TCTN1 was a direct target of miR-216a-5p. **a** Putative binding sites of 216a-5p within the 3′-UTR region of TCTN1 mRNA, and the sequences of wild-type and mutant-type vector. **b** The relative luciferase activities were inhibited in 293 T cells co-transfected with wild-type TCTN1 3′-UTR vector and miR-216a-5p, not with the mutant-type vector. Firefly luciferase activity was normalized to Renilla luciferase. Data are presented as the mean value ± SD from triplicate experiments. ***p < 0.001 vs. miR-NC; **c** mRNA and **d** protein levels of TCTN1 were detected by qRT-PCR and western blot in EC9706 and TE-9 cells transfected with miR-216a-5p or miR-NC. **e** The protein level of TCTN1 was determined by western blot in HET-1A cells transfected with miR-216a-5p inhibitor or miR-NC. **f** Relative expression levels of TCTN1 mRNA in ESCC tissues and adjacent tissues were detected by qRT-PCR. **g** Pearson’s correlation analysis for the relationship between miR-216a-5p levels and TCTN1 mRNA levels in ESCC tissues

To reveal the molecular mechanisms underlying the roles of miR-216a-5p on ESCC cell proliferation and apoptosis, online bioinformatics analysis was performed to predict the potential target genes of miR-216a-5p. As presented in Fig. 3a, the 3′-UTR of TCTN1 was found to contain a complementary region of miR-216-5p seed sequences, indicating that TCTN1 might be a potential target gene of miR-216-5p. Subsequently, a luciferase report assay was performed to examine whether miR-216a-5p may directly target TCTN1. The results revealed that introduction of miR-216a-5p to 293 T cells could significantly reduce the luciferase activity of the reporter plasmid with WT TCTN1 but did not obviously change the expression of a reporter fused to MUT TCTN1 (Fig. 3b, *p* < 0.001). Moreover, qRT-PCR (Fig. 3c, *p* < 0.001) and western blot (Fig. 3d) analyses further confirmed that miR-216a-5p overexpression reduced TCTN1 expression at mRNA and protein levels in both EC9706 and TE-9 cells. In addition, we found that silencing miR-216a-5p in the normal esophageal epithelial cell line HET-1A by inhibitor upregulated the expression of TCTN1 (Fig. 3e). Next, we measured mRNA expressions of TCTN1 in ESCC tissues and the matched adjacent specimens. As shown in Fig. 3f, the expression of TCTN1 was significantly higher in ESCC tissues compared with adjacent normal tissues. Pearson’s correlation analysis further demonstrated that the expression levels of miR-216a-5p and TCTN1 were inversely correlated in ESCC tissues (Fig. 3g, *p* = 0.0425). These data implied that TCTN1 might play important roles in ESCC, which was directly targeted by miR-216a-5p.

### TCTN1 knockdown inhibited cell proliferation and induced apoptosis in ESCC

**Fig. 4:**
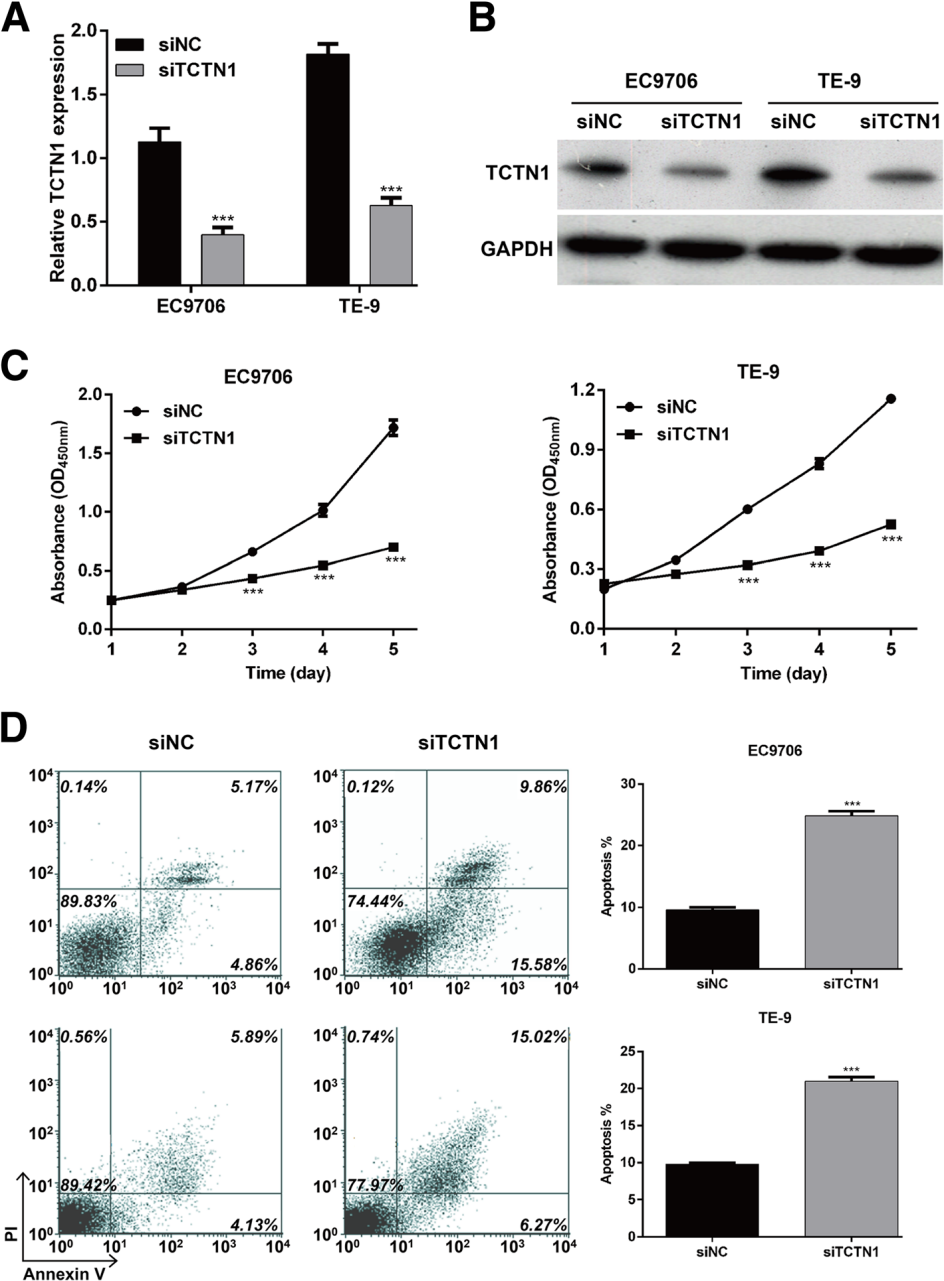
TCTN1 knockdown inhibited cell proliferation and induced apoptosis in ESCC. EC9706 and TE-9 cells were transfected with siTCTN1 or siNC, respectively. **a** qRT-PCR and **b** western blot analysis were performed to measure the TCTN1 expression at mRNA and protein levels, respectively. **c** CCK-8 assay was used to analyze cell proliferation in EC9706 and TE-9 cells. **d** Flow cytometry with double Annexin V/PI staining was used to detect the ESCC cell apoptosis. ***p < 0.001 vs. siNC

We next investigated whether miR-216a-5p affected cell proliferation and apoptosis of ESCC cells by targeting TCTN1. Both EC9706 and TE-9 cells were transfected with siTCTN1 to decrease the expression of TCTN1, as confirmed by qRT-PCR (Fig. 4a, *p* < 0.001) and western blot analysis (Fig. 4b). The CCK-8 assay revealed that the cell proliferation rate was remarkably impaired in siTCTN1 transfected EC9706 and TE-9 cells compared with siNC groups (Fig. 4c, *p* < 0.001). Consistently, TCTN1 knockdown significantly increased the overall apoptotic percentage from 9.58% ± 0.44 to 24.84% ± 0.74% in EC9706 and from 9.79% ± 0.21 to 20.98% ± 0.58% in TE-9 cells (Fig. 4d, *p* < 0.001). These data indicated that TCTN1 down-regulation might be the mechanism of the miR-216a-5p induced decreased proliferation and increased apoptosis of ESCC cells.

### Restoration of TCTN1 abolished the effects of miR-216a-5p on cell proliferation and apoptosis

**Fig. 5:**
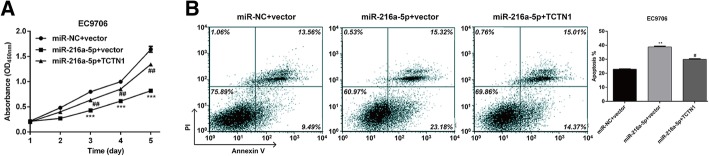
Addition of TCTN1 reversed miR-216a-5p-mediated effects on cell proliferation and apoptosis. EC9706 cells were co-transfected with miR-216a-5p mimic/miR-NC and with TCTN1 overexpression plasmid/empty vector. **a** CCK-8 assay was used to analyze cell proliferation. **b** Flow cytometry with double Annexin V/PI staining was used to detect cell apoptosis. **p < 0.01, ***p < 0.001 vs. siNC+vector; #*p* < 0.05, ##*p* < 0.01 vs. miR-216a-5p + vector

**Fig. 6:**
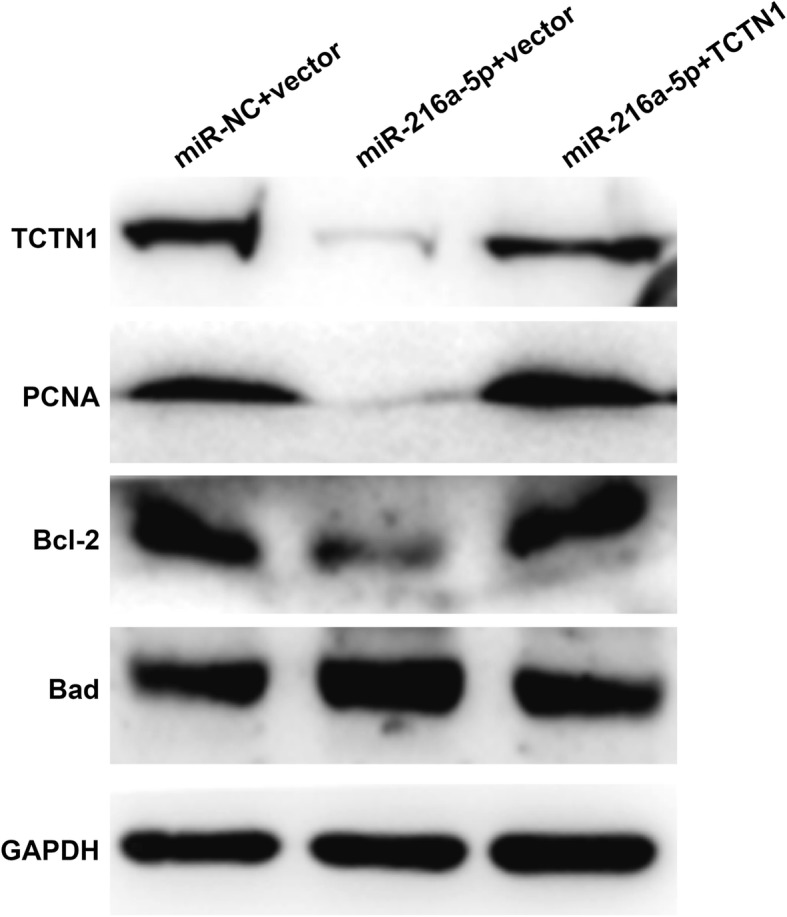
Addition of TCTN1 reversed the regulation of miR-216a-5p on the proliferation and apoptotic markers in ESCC. EC9706 cells were co-transfected with miR-216a-5p mimic/miR-NC and with TCTN1 overexpression plasmid/empty vector. Western blot analysis was performed to analyze the expression of PCNA, Bcl-2 and Bad in EC9706 cells

To further explore whether TCTN1 was required for the miR-216a-5p-mediated effects on cell proliferation and apoptosis, we next performed rescue experiments by transfecting an overexpression plasmid of TCTN1 (pcDNA3.1-TCTN1) into EC9706 cells treated with miR-216a-5p. As expected, the decreased cell proliferation and increased cell apoptosis by miR-216a-5p were partially abolished by overexpressing TCTN1 in EC9706 cells, as determined by CCK-8 (Fig. 5a) and flow cytometry analysis (Fig. 5b), respectively. Furthermore, we detected the expression alterations of several markers. As shown in Fig. 6, miR-216a-5p overexpression obviously down-regulated TCTN1 expression, leading to a reduction of PCNA, anti-apoptotic protein Bcl-2 and elevation of pro-apoptotic protein Bad. However, TCTN1 overexpression reversed the effects of miR-216a-5p transfection on the expression of TCTN1, PCNA, Bcl-2 and Bad. These findings further demonstrated that TCTN1 might be a key regulator in the miR-216a-5p-mediated cell proliferation and apoptosis in ESCC cells.

## Discussion

In the present study, we found that overexpression of miR-216a-5p in ESCC cells obviously suppressed cell proliferation and induced apoptosis. Meaningfully, we could link changes in cell growth and survival to the direct targeting of TCTN1, which participated in a diverse range of developmental procedures [27].

The miR-216a-5p gene was previously identified only in pancreatic ductal adenocarcinoma [14] and renal cell carcinoma. Interestingly, miR-216a-5p has dual roles in regulating cancer cell development and tumorigenesis, which can inhibit pancreatic ductal adenocarcinoma cell proliferation, migration and invasion [25]. By contrast, Chen et al. [16] pointed to the oncogenetic role of miR-216a-5p in renal cancer. Considering that the role of miR-216a-5p is distinct in different tumor types, the expression of miR-216a-5p was first determined individually in ESCC tissues and cell lines. Here, we found that miR-216a-5p was significantly down-regulated in ESCC tissues and cell lines. EC9706 and TE-9 cell lines were chosen for subsequent loss-of-function analysis. The results showed that proliferation in ESCC cells can be blocked and apoptosis can be stimulated by miR-216a-5p, suggesting an anti-tumor role of miR-216a-5p in ESCC.

Degradation of miR-216a-5p targets is the dominant effect of miR-216a-5p in cancer, including (i) inhibition of hexokinase-2 in uveal melanoma [28] and (ii) down-regulation of matrix metalloproteinase 16 in lung cancer [15]. In particular, TCTN1 was regarded as a possible target gene of miR-216a-5p by using TargetScan. Moreover, luciferase reporter assay confirmed this prediction as the relative luciferase activities were suppressed in 293 T cells co-transfected with WT TCTN1 3′-UTR vector and miR-216a-5p, compared with mutant-type vector. Generally, the biological behaviors of ESCC cells with knockdown of TCTN1 tended to imitate these cells with overexpression of miR-216a-5p. Mechanistically, we suggest that miR-216a-5p exerts its tumor suppressive role via targeting TCTN1. Notably, overexpression of miR-216a-5p could down-regulate the expression of PCNA and pro-apoptotic Bad, but up-regulate the expression of anti-apoptotic Bcl-2, which was reversed by TCTN1 overexpression.

Numerous growth pathways contribute to cell proliferation in cancer cells [29]. PCNA, a ring-shaped protein, is also known as a critical component of the DNA machinery responsible for DNA replication and genomic stability [30, 31]. It exerts biological effects on DNA damage repair and DNA proliferation through binding to the flap endonuclease 1 (FEN-1) and xeroderma pigmentosum (XP) G, and facilitates resynthesis of a new DNA fragment [32, 33]. In recent years, many studies have provided a deeper comprehension of PCNA as a factor in miRNA-mediated regulation of cellular development, growth and maintenance. MiR-363-3p has been reported to suppress the specific target gene PCNA to exert an anti-proliferation effect on lung cancer cells [34]. The anti-tumor activity of miR-149 in glioma cells was found to be correlated with low-expression of PCNA, p-AKT1, cyclin D1, and MMP-2 [35]. In this study, we suggest that, with the enhanced expression of miR-216a-5p, signaling to proliferation can be inactivated due to the down-regulated expression of PCNA.

Beneath the complexity and heterogeneity of each cancer lies survival events that have propelled the cancer cells escaped from pathognomonic cellular changes [36]. Accumulating evidence indicates that the processes of neoplastic transformation, development and tumorigenesis involve abnormalities in apoptosis signaling pathways [37]. In our present study, overexpressing miR-216a-5p ESCC cells showed a significant decrease in PCNA and Bcl-2 and an increase in Bad. Bcl-2, an inner mitochondrial transmembrane protein, is known to be a key anti-apoptotic regulator [38]. The sensitizer BH-3-only protein Bad could induce apoptosis by binding and inactivating Bcl-2 and anti-apoptotic Bcl-xL [39, 40]. Current understanding of molecular ESCC apoptosis suggests that up-regulation of Bcl-2 and down-regulation of Bad caused by TCTN1 overexpression are major mechanisms of miR-216a-5p-mediated apoptosis.

These findings lead to the interesting conclusion that inhibition of ESCC cell carcinogenesis occurred in response to miR-216a-5p targeting TCTN1 characterized by anti-proliferative activity and induction of apoptosis. In tumor cells, the expression of TCTN1 has been demonstrated to be overexpressed in many different cancer types [23, 26, 41]. Silencing of TCTN1 was suggested to induce human thyroid cancer cell apoptosis through over-expression of cleaved caspase-3 and PARP and repression of Bcl-2 [41]. Silencing of TCTN1 by lentivirus-mediated RNA interference in gastric cancer and pancreatic cancer cells and reduction of proliferation were observed, suggesting that the knockdown of TCTN1 is sufficient to inhibit cell viability [23, 42]. In this study, the expression levels of TCTN1 were up-regulated in ESCC tissues compared with adjacent tissues. Further data confirmed that miR-216a-5p suppressed ESCC cell proliferation at least partially by down-regulating TCTN1 expression.

## Conclusions

In conclusion, our findings identify the miR-216a-5p/TCTN1 axis as a mechanism of miR-216a-5p-mediated inhibition of ESCC cell proliferation and induction of apoptosis, with potentially important implications for development of ESCC therapy.

## Data Availability

The data in this study are available from the author for correspondence upon reasonable request.

## Abbreviations

ESCC: Esophageal squamous cell carcinoma
miRNA: MicroRNA
qRT-PCR: Quantitative real-time PCR
TCTN1: Target tectonic family member 1
UTR: Untranslated region
